# Sensory phenomena in children with Tourette syndrome or autism spectrum disorder

**DOI:** 10.3389/fpsyt.2024.1338234

**Published:** 2024-04-02

**Authors:** Adriana Prato, Federica Saia, Marianna Ferrigno, Valentina Finocchiaro, Rita Barone, Renata Rizzo

**Affiliations:** Child and Adolescent Neurology and Psychiatric Section, Department of Clinical and Experimental Medicine, Catania University, Catania, Italy

**Keywords:** Tourette syndrome, autism spectrum disorder, neurodevelopmental disorders, children, sensory phenomena

## Abstract

**Background:**

Tourette syndrome (TS) and autism spectrum disorder (ASD) are two neurodevelopmental disorders with an onset before the age of 18 years. TS patients frequently reported atypical sensory phenomena (SP). Sensory processing abnormalities are also particularly frequent in ASD individuals.

**Objectives:**

Considering the higher rate of atypical sensory behaviours in both neurodevelopmental disorders, in the present study we analysed sensory experiences in patients with ASD and in patients with TS.

**Methods:**

We enrolled patients with a primary diagnosis of TS or ASD. All participants were assessed for primary diagnosis and associated comorbidities. The presence of sensory behaviours was investigated using the University of Sao Paulo’s Sensory Phenomena Scale (USP-SPS).

**Results:**

SP were significantly more represented in the ASD-group versus TS-group, except for sound just-right perceptions and energy to released. ASD participants presented higher mean scores in all fields of USP-SPS severity scale respect on TS patients and healthy controls. The USP-SPS total score had significant positive correlations with the CYBOCS and MASC total scores in the TS cohort. In the ASD group, the USP-SPS total score was significantly negative correlated with the total IQ and marginally positive correlated with ADOS total score.

**Conclusion:**

SP are a frequently reported characteristic both of ASD and TS. Future studies are needed to better evaluate the differences on their phenomenology in patients with TS and ASD.

## Introduction

Tourette syndrome (TS) is a neurodevelopmental disorder most diagnosed in childhood or early adolescence, characterized by multiple motor tics and/or vocal tics, which last for more than 12 months, with an onset age before 18 years (1). The reported prevalence of TS was even estimated to be 0.3-1% (2). Patients affected by TS frequently report a range of comorbid psychopathologies, such us attention deficit hyperactivity disorder (ADHD), obsessive-compulsive disorder (OCD), autism spectrum disorder (ASD), anxiety disorders and sleep disorders (3, 4). Individuals with TS experience a variety of different sensory phenomena (SP), including premonitory urges prior to tics, “just right” perceptions, or somatic hypersensitivity due to impaired sensorimotor gating (5). For this reason, sensory phenomena are recognized as core TS symptoms.

ASD is a childhood-onset neurodevelopmental disorder, characterized by significant defects of social communication and interaction across multiple contexts, associated with restricted and repetitive patterns of interests and activities (1). The reported global prevalence of ASD was approximately estimates to be 1% (6) and most recent of about 2% in the United States (US) (7). Difficulty processing, integrating, and responding to sensory stimuli has been reported as a characteristic of ASD since the first report of this neurodevelopmental condition (8). Indeed, in the fifth edition of the Diagnostic and Statistical Manual of Mental Disorders (DSM-5), sensory reactivity symptoms were associated to the restricted and repetitive behaviour domain, as a diagnostic criterion (9). Recent estimates reported that between 45 and 96% of children with ASD manifest these sensory difficulties (10, 11).

TS and ASD frequently co-occur and both present similar clinical and behavioural features (12). The reported prevalence of comorbid ASD in subject affected by TS is variable, ranging from 2.9% to 20% (13–15). Abnormalities in corticostriatal circuits are common in both disorders, that are etiologically related (16 No more studies have explored the differences between SP in TS and ASD. The present study aimed to evaluate sensory behaviours in two large populations from a single center, TS patients and ASD patients, compared with a pediatric control group. Specifically, we used the University of Sao Paulo’s Sensory Phenomena Scale (USP-SPS) to (1) examine how children with ASD or TS attend to sensory stimuli, (2) assess sensory experiences both in patients with ASD and in patients with TS, differentiating their types, (3) evaluate the reliability of the USP-SPS especially in the ASD sample.

## Materials and methods

### Study design

This study was performed at the Child and Adolescent Neurology and Psychiatry, Department of Clinical and Experimental Medicine, Catania University. Participants comprised 92 children with the TS diagnosis, 82 children with the ASD diagnosis and 100 typically developing (TD) controls, with a similar age and gender distribution as the patients. All participants underwent a full neuropsychiatric assessment by a team of child and adolescent neurologists with a specific expertise in the evaluation of neurodevelopmental conditions. The study was conducted in agreement with the Declaration of Helsinki and authorized by the local Ethics Committee of Catania University Hospital. The informed consent of the children’s and their parents involved in the study was obtained to enter clinical and demographic data from the clinical files into this study.

### Participants

Eligible participants were children aged 5-17 years that presented a primary diagnosis of TS or ASD based on DSM-V criteria (1). We excluded patients older than 18 years, who presented a moderate or severe intellectual disability, or other primary psychiatric disorders, different from TS or ASD. Comorbidity with other neuropsychiatric disorders was not established as an exclusion criterion if TS or ASD were the primary diagnosis. As a control group (n=100) we included subjects with typical development (TD) from a community sample with no neurodevelopmental disturbances and with an age and gender distribution equal to the patients with ASD or TS. TD participants’ exclusion criteria included positive history for intellectual disability or other developmental, neurological, or behavioural problems. The Social Communication Questionnaire (SCQ) (17) was used to screen and exclude autism in TD children.

### Clinical assessment

The clinical assessment of our sample was conducted by paediatric neuropsychiatrist with solid experience in developmental disorders. Participants underwent assessment of intelligence quotient using the Wechsler Intelligence Scale for Children (WISC-IV) (18). The clinical symptoms of TS and ASD patients were evaluated through the administration of the Yale Global Tic Severity Rating Scale (YGTSS), Children’s Yale-Brown Obsessive-Compulsive Scale for Children (CY-BOCS), Autism Diagnostic Observation Schedule (ADOS), Multidimensional Anxiety Scale for Children (MASC), Child Depression Inventory (CDI), Conners’ Parent Rating Scale (CPRS) and Child Behaviour Checklist (CBCL). In addition, the USP-SPS was performed to assess the presence and severity of sensory behaviours.

### Measures

The YGTSS is a clinician-rated instrument administered to evaluate the motor and phonic tic severity. This scale presents two separate motor and vocal tic checklists scored from 0 to 5 on two subscales for motor and vocal tics, also combined to obtain a total tic severity score. Another score ranging from 0 to 50 was calculated for global impairment due to tic symptoms (19). To evaluate OCD, the CY-BOCS, a semi-structured clinician-administered interview evaluating the severity of obsessions and compulsions was also conducted (20). The ADOS was used for ASD diagnosis. The ADOS is a direct observation that consists of four modules of exploration (A), social interaction (B), imagination (C), and repetitive and stereotyped behaviours (D) (21). The CPRS is a practical instrument for acquiring parental reports of childhood behaviour problems that contains summary scales supporting ADHD diagnosis and quantifying ADHD severity (22). The CBCL is a very useful questionnaire administered to assess a variety of behavioural and emotional problems (anxiety, depression, introversion) in the children (23). The CDI is a self-report tool that evaluate depressive symptoms in children and adolescents (24). All participants also completed the MASC, a standardized measure of anxious symptoms (25). In addition, the presence and severity of sensory phenomena was evaluated through the administration of the USP-SPS, a semi structured scale that contain a checklist and a severity scale (26). The USP-SPS checklist evaluate the occurrence of possible different subtypes of SP including physical sensations, “just-right” perceptions, feelings of incompleteness, energy that builds up and needs to be released, and just an urge to do repetitive behaviours. The USP-SPS severity scale measures the severity of the SP considering the frequency of symptoms, the amount of distress that they determined, and the degree to which they interfere with patient’s quality of life (26).

### Statistical analysis

Data were analyzed using SPSS software (SPSS, Inc., Chicago, IL, USA, IBM, Somers, NY, USA). Continuous variables were reported as mean (standard deviation), while categorical variables were reported as absolute values (n) and relative values (%). The distribution of quantitative data was normality assessed by the Shapiro-Wilk test. Student’s t tests were conducted to compare clinical variables and rating scales between ASD and TS groups. Pearson’s chi-square tests were performed to compare categorical variables between ASD and TS, and between TD subjects for the evaluation of SP. In addition, Pearson’s correlation coefficients were determinate to investigate the correlation between the total USP-SPS score and other scale scores. A p-value < 0.05 was considered to reveal statistical significance.

## Results

### Sample characteristics

In this study, we enrolled a clinical cohort of 274 individuals aged 5-17 years (mean age = 10.4 ± 2.6; male (M)/female (F) = 183:91; male = 66.8%). Of the entire cohort, 92 subjects were affected by TS, 82 patients presented a diagnosis of ASD. Participants comprised also 100 TD subjects, with a similar age and gender distribution as the patients (75 males, 25 females; mean age 9.5 ± 0.6). TS patients were 70 males and 22 females, with a mean age of 10.65 ± 2.8 years. The mean age of tic onset was 6.6 (± 2.1) years, while the mean age of the diagnosis was 8.8 (± 2.5) years. Of the 92 patients affected by TS, 35 subjects (38.04%) had a family history of TS, 33 subjects (35.09%) had a family history of OCD, 6 subjects (6.5%) had a family history of ADHD, and another 20 (21.7%) had a family history of depression. Among the individuals affected by TS, the most common neuropsychiatric comorbidities were OCD (64.1%) and conduct disorder – CD (26.1%); 9.8% of the TS-affected participants also met the diagnostic criteria for ADHD. Only 24 patients (26.1%) presented “pure-TS” phenotype; conversely, 68 patients (73.9%) presented also associated comorbidities, in particular one (n=46), two (n=18) or more (n=4) comorbid disorders. Patients with ASD (n= 82) included 71 males and 11 females, with a mean age of 11.2 ± 3.4. The mean age of symptoms ‘onset was 2.76 ( ± 0.8) years, while the mean age of the diagnosis was 5.2 (± 2.3) years. Of the 82 patients affected by ASD, 8 subjects (9.8%) had a family history of OCD, 9 subjects (10.98%) had a family history of ADHD, and another 9 (10.98%) had a family history of depression. None of the ASD cohort reported a family history of TS. Considering ASD sample, 57 patients (69.5%) presented associated comorbidities, in particular one (n=23), two (n=18) or more (n=16) comorbid disorders. 41.5% of the ASD-affected participants had a comorbid OCD, 37.8% had a comorbid CD, and another 28.05% a comorbid ADHD. Demographic data and clinical features of all participants are displayed in **Table 1**. Compared with TS, patients with ASD were younger at symptom onset (mean age 2.76 vs 6.6, t = 15.269, p < 0.00001) and at the time of diagnosis (mean age 5.2 vs 8.8, t = 10.08, p < 0.00001) (**Table 1**). Participants with ASD were more likely to have echolalia (74.4% vs 10.9%, χ²(df) = 72.425, p < 0.00001), self-injurious behaviours (13.4% vs 2.2%, χ²(df) = 7.924, p = 0.0049), and a comorbid diagnosis of ADHD (28.05% vs 9.8%, χ²(df) = 9.638, p = 0.0019) or sleep disorders (24.4% vs 3.3%, χ²(df) = 16.874, p = 0.00004) (**Table 1**). Conversely, TS patients were more likely to have a positive family history for tics (38.04% vs 1.2%, χ²(df) = 35,829, p < 0.00001) or OCD (35.9% vs 9.8%, χ²(df) = 16,415, p = 0.00005), and a comorbid diagnosis of OCD (64.1% vs 41.5%, χ²(df) = 8.953, p = 0.0028) (**Table 1**). Compared to ASD participants, TS patients were more likely to have a single comorbid diagnosis (50.0% vs. 28.05%, χ²(df) = 8,73, p = 0.0031). Conversely, participants with ASD were more likely to have ≥ 3 associated comorbidities (19.5% vs. 4.3%, χ²(df) = 9,801, p = 0.0017). Instead, there was no significant difference between the TS group and the ASD group considering the other clinical and demographic variables (**Table 1**).

**Table 1:** Demographic and clinical features of the participants.

Partecipant characteristics	Total sample (n=174)	ASD (n=82)	TS (n=92)	p-value
Male (%)	141 (81.0%)	71 (86.6%)	70 (76.1%)	0.078
Mean age (years) ± SD	10.4 (± 2.6)	11.2 (± 3.4)	10.65 (± 2.8)	0.2287
Age of onset (mean ± SD)	4.8 (± 2.5)	2.76 (± 0.8)	6.6 (± 2.1)	< 0.00001
Age of diagnosis (mean ± SD)	7.1 (± 2.99)	5.2 (± 2.3)	8.8 (± 2.5)	< 0.00001
Echolalia	71 (40.8%)	61 (74.4%)	10 (10.9%)	< 0.00001
Coprolalia	5 (2.9%)	1 (1.2%)	4 (4.3%)	0.2176
Palilalia	7 (4.0%)	3 (3.66%)	4 (4.3%)	0.817
Self-injurious behaviors	13 (7.5%)	11 (13.4%)	2 (2.2%)	0.0049
Family history (n, %)				
TS	36 (20.7%)	1 (1.2%)	35 (38.04%)	< 0.00001
OCD	41 (23.6%)	8 (9.8%)	33 (35.9%)	0.00005
ADHD	15 (8.6%)	9 (10.98%)	6 (6.5%)	0.296
Depression	29 (16.7%)	9 (10.98%)	20 (21.7%)	0.057
**Comorbid diagnosis (n, %)**	125 (71.8%)	57 (69.5%)	68 (73.9%)	0.519
+ 1 comorbid diagnosis	69 (39.66%)	23 (28.05%)	46 (50.0%)	0.0031
+ 2 comorbid diagnosis	36 (20.7%)	18 (21.95%)	18 (19.6%)	0.698
≥ 3 comorbid diagnosis	20 (11.5%)	16 (19.5%)	4 (4.3%)	0.0017
Comorbid diagnosis (n, %)				
OCD	93 (53.4%)	34 (41.5%)	59 (64.1%)	0.0028
ADHD	32 (18.4%)	23 (28.05%)	9 (9.8%)	0.0019
CD	55 (31.6%)	31 (37.8%)	24 (26.1%)	0.097
Sleep disorders	23 (13.2%)	20 (24.4%)	3 (3.3%)	0.00004

SD, standard deviation. ASD, Autism Spectrum Disorder; TS, Tourette Syndrome; OCD, Obsessive-Compulsive Disorder; ADHD, Attention-deficit hyperactivity disorder; CD, conduct disorder. p-values refer to Pearson’s chi-square tests in case of categorical variables (summarized by absolute and percent frequencies), and to Student’s t tests in case of quantitative variables (summarized by means and SD).

### Neuropsychiatric evaluation

The results of the neuropsychiatric evaluation are summarized in **Table 2**. TS patients compared to ASD patients presented significantly higher mean total IQ (total IQ: mean 93.6, SD ± 18.2 vs. mean 86.1, SD 21.4, t = 2.4853, p = 0.00695) (**Table 2**). Participants with TS presented a mean total YGTSS score of 17.4 (± SD 9.4). Instead, evaluation through ADOS-2 in ASD patients showed total ASD score (Social Affect +Restricted and repetitive behaviours) of 10,7 (± SD 4.0). The mean scores for CY-BOCS were statistically significant higher in TS patients (total CY-BOCS: mean 13.9, SD ± 8.4 vs. mean 9.1, SD ± 5.8, t = 4.3427, p < 0.00001) (**Table 2**). No statistically significant differences were also observed between the two groups based on total CDI (p = 0.246) and MASC scores (p = 0.141) (**Table 2**). The comparison between the mean CBCL scores in the two clinical groups showed statistically significant differences for total scores (total CBCL score: mean 46.8, SD ± 20.5 vs. mean 38.9, SD ± 24.5, t= - 2.3033, p = 0.0112) and “internalizing problems” (mean 13.7, SD ± 6.6 vs. mean 11.3, SD ± 9.6, t= - 1.8544, p = 0.0327); in contrast, the mean scores for “externalizing problems” were not statistically significant different (p = 0.4537) (**Table 2**). Furthermore, the two cohorts presented non-statistically significant different scores in all fields of CPRS, except for “ADHD index” (total “ADHD index”: mean 9.75, SD ± 8.7 vs. mean 4.1, SD 2.9, t = - 2.9571, p = 0.0018) (**Table 2**).

**Table 2:** Neuropsychiatric evaluations of TS and ASD participants.

Measures	ASD (n=82)	TS (n=92)	p-value
IQ			
TIQ	86.1 (± 21.4)	93.6 (± 18.2)	0.00695
VIQ	87 (± 23.1)	95 (± 19.1)	0.0063
PIQ	88.1 (± 21.4)	94.1 (± 17.2)	0.0207
YGTSS			
Total	3.7 (± 4.9)	17.4 (± 9.4)	<0.00001
Motor	2.9 (± 3.7)	10.8 (± 5.4)	<0.00001
Phonic	0.8 (± 2.2)	6.4 (± 5.4)	<0.00001
CY-BOCS			
Total	9.1 (± 5.8)	13.9 (± 8.4)	<0.00001
Obsessions	4.9 (± 3.3)	7.2 (± 4.4)	0.0001
Compulsions	4.1 (± 2.9)	6.7 (± 4.8)	0.000015
CPRS			
Total	30.7 (± 16.4)	27.1 (± 20.4)	0.1054
Oppositional problems	6.1 (± 3.6)	6.6 (± 5.2)	0.2441
Cognitive Problems/Inattentive	6.2 (± 4.4)	5.7 (± 4.96)	0.2461
Hyperactivity-Impulsivity	4.6 (± 3.8)	5.8 (± 8.5)	0.1186
ADHD index	4.1 (± 2.9)	9.75 (± 8.7)	0.0018
**CDI**	10.1 (± 6.4)	9.4 (± 7.1)	0.246
MASC			
Total	44.4 (± 13.4)	42.2 (± 14.0)	0.141
Physical symptoms	11.7 (± 4.97)	11.6 (± 5.3)	0.4795
Harm avoidance	14.66 (± 4.5)	13.9 (± 5.96)	0.186
Separation anxiety/phobias	10.0 (± 4.95)	10.2 (± 5.6)	0.3775
CBCL			
Total	46.8 (± 20.5)	38.9 (± 24.5)	0.0112
Internalizing problems	13.7 (± 6.6)	11.3 (± 9.6)	0.0327
Externalizing problems	16.26 (± 9.9)	16.05 (± 12.6)	0.4537
ADOS			
Social affect (SA)	7.7 (± 2.3)	0.02 (± 0.1)	<0.00001
Restricted and Repetitive Behaviors (RRB)	3.0 (± 1.7)	1.5 (± 1.2)	<0.00001
SA + RRB	10.7 (± 4.0)	1.52 (± 1.3)	<0.00001

ASD, Autism Spectrum Disorder; TS, Tourette Syndrome; IQ, Intelligence quotient; TIQ, Total Intelligence quotient; VIQ, Verbal Intelligence quotient; PIQ, Performance Intelligence Quotient; YGTSS, Yale Global Tic Severity Rating Scale; CY-BOCS, Children’s Yale-Brown Obsessive-Compulsive Scale for Children; CPRS, Conners’ Parent Rating Scale; ADHD, Attention-deficit hyperactivity disorder; CDI, Child Depression Inventory; MASC, Multidimensional Anxiety Scale for Children; CBCL, Child Behaviour Checklist; ADOS, Autism Diagnostic Observation Schedule; SA, Social affect; RRB, Restricted and Repetitive Behaviors. p-values refer to Student’s t tests conducted to compare rating scale between ASD and TS groups (summarized by means and SD).

### Evaluation of sensory phenomena

**Figure 1:**
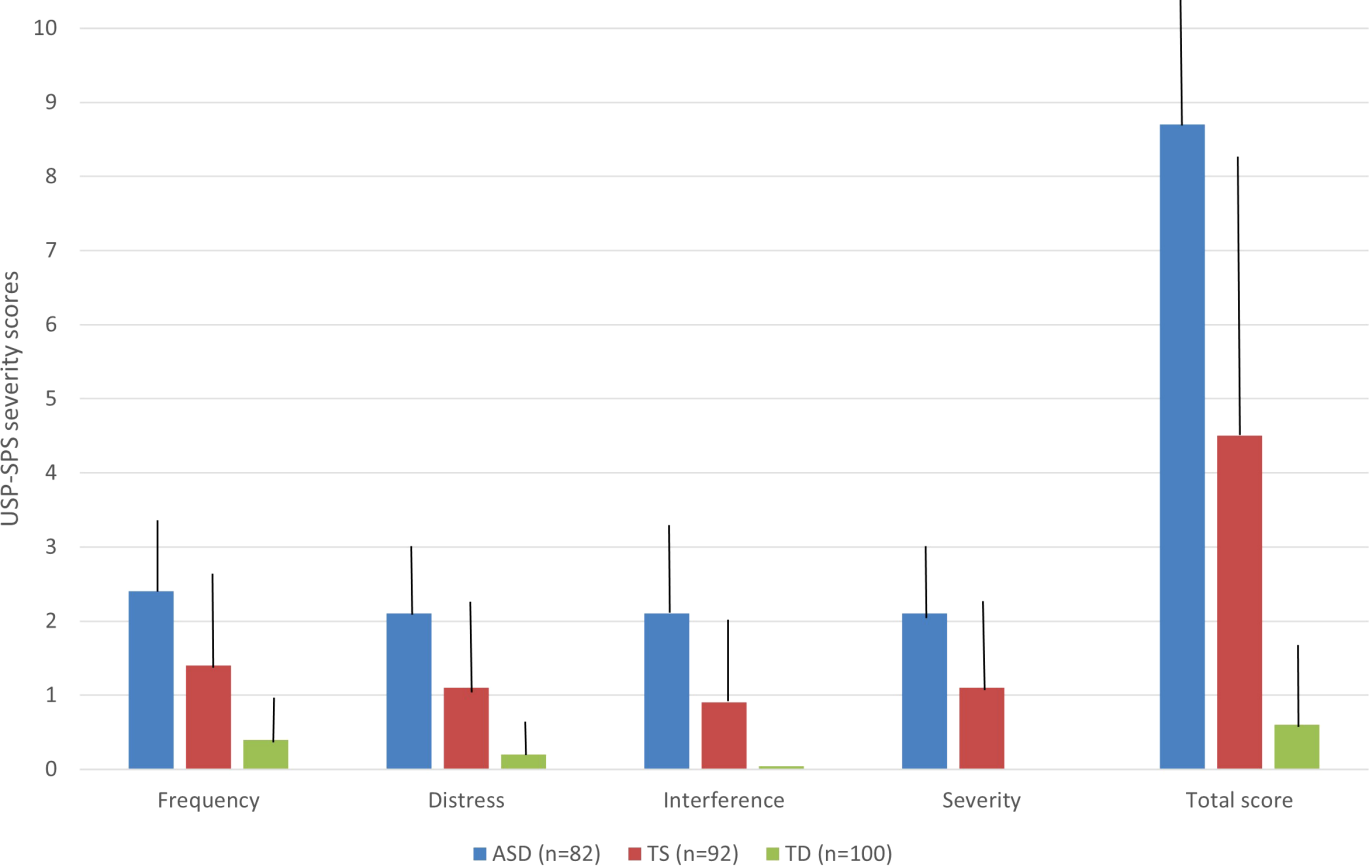
Severity scores of sensory phenomena (SP). The figure reports the results of USP-SPS total severity scores across the three groups (ASD in blue, TS in red, and TD in green). Bars indicate the standard deviations; all fields of USP-SPS severity scale were significantly higher in ASD-group respect on TS-group and TD-group.

All participants of the entire cohort (n = 274), also including a control group, completed the USP-SPS to evaluate the presence and severity of different types of SP. All 82 participants affected by ASD experienced some SP. SP were present also in 76 TS patients (82.6%) and 31 TD subjects (31%) (**Table 3**). As for types of SP in ASD cohort, 81 participants (98.8%) presented hypersensitivity, followed by tactile physical sensations (n = 68, 82.9%) and look “just-right” perceptions (n = 62, 75.6%) (**Table 3**). In the TS cohort, 54 patients (58.7%) referred tactile physical sensations, followed by look “just-right” perceptions (n = 47, 51.1%) (**Table 3**). Furthermore, 31 TD subjects (31%) experienced some SP, frequently look “just-right” perceptions (n = 22, 22%) and tactile physical sensations (n = 17, 17%) (**Table 3**). Statistically significant differences were detected based on all subtypes of SP in the TS-group versus the ASD-group, with some exceptions. Furthermore, all subtypes of SP were significantly more represented in the ASD-group versus TS-group, except for sound just-right perceptions (32.9% vs 29.35%, χ²(df) = 0.259, p = 0.6105) and energy to released (34.1% vs 25%, χ²(df) = 1.751, p = 0.186). All subtypes of SP were also significantly more represented in the ASD group versus TD group, and in TS group versus TD group (**Table 3**). The mean USP-SPS total severity scores for all groups are displayed in **Figure 1**. In the current study, the ASD clinical cohort had a significantly higher mean USP-SPS total score than that of the TS patients (mean USP-SPS total score: 8.7 vs 4.5, t = -8.40554, p < 0.00001). ASD participants presented also higher mean scores in all fields of USP-SPS severity scale respect on TS patients and TD subjects (p< 0.00001) (**Table 4**).

**Table 3:** Assessment of sensory phenomena (SP) through USP-SPS checklist.

USP-SPS	Current	ASD (n=82)	Current	TS (n = 92)	Current	TD (n=100)	ASD vs TS	ASD vs TD	TS vs TD
		Previous/Absence		Previous/Absence		Previous/Absence	p-value	p-value	p-value
**Presence of any SP**	82 (100%)	0 (0%)	76 (82.6%)	16 (17.4%)	31 (31%)	69 (69%)	<0.0001	<0.0001	< 0.0001
Subtypes of SP									
Tactile Physical Sensation	68 (82.9%)	14 (17.1%)	54 (58.7%)	38 (41.3%)	17 (17%)	83 (83%)	0.0005	<0.0001	<0.0001
Muscle-joint or bone Physical Sensations	39 (47.6%)	43 (52.4%)	28 (30.4%)	64 (69.6%)	1 (1%)	99 (99%)	0.0205	<0.0001	<0.0001
Look just-right perception	62 (75.6%)	20 (24.4%)	47 (51.1%)	45 (48.9%)	22 (22%)	78 (78%)	0.0008	<0.0001	0.00003
Sound just-right perception	27 (32.9%)	55 (67.1%)	27 (29.35%)	65 (70.65%)	3 (3%)	97 (97%)	0.6105	<0.0001	<0.0001
Feel just-right perception	43 (52.44%)	39 (47.6%)	21 (22.8%)	71 (77.2%)	2 (2%)	98 (98%)	0.00005	<0.0001	<0.0001
Feeling of incompleteness	30 (36.58%)	52 (63.4%)	15 (16.3%)	77 (83.7%)	1 (1%)	99 (99%)	0.0002	<0.0001	0.00013
Energy to released	28 (34.1%)	54 (65.9%)	23 (25%)	69 (75%)	0 (0%)	100 (100%)	0.186	<0.0001	<0.0001
Urge to do repetitive behaviours	37 (45.1%)	45 (54.9%)	20 (21.74%)	72 (78.3%)	0 (0%)	100 (100%)	0.0010	<0.0001	<0.0001
Hypersensitivity	81 (98.8%)	1 (1.2%)	14 (15.2%)	78 (84.8%)	0 (0%)	100 (100%)	<0.0001	<0.0001	<0.0001

ASD, Autism Spectrum Disorder; TS, Tourette Syndrome; TD, typically developing; USP-SPS, University of Sao Paulo’s Sensory Phenomena Scale; SP, sensory phenomena; p-values refer to Pearson’s chi-square tests to compare categorical variables between ASD, TS and TD groups (summarized by absolute and percent frequencies).

**Table 4:** Assessment of sensory phenomena (SP) through USP-SPS severity scale.

USP-SPS severity scale	ASD (n=82)	TS (n=92)	TD (n= 100)	p (ASD vs TS)	p (ASD vs TD)	p (TS vs TD)
**Frequency**	2.4 (± 0.9)	1.4 (± 1.3)	0.4 (± 0.6)	< 0.00001	< 0.00001	< 0.00001
**Distress**	2.1 (± 0.9)	1.1 (± 1.1)	0.2 (± 0.4)	< 0.00001	< 0.00001	< 0.00001
**Interference**	2.1 ( ± 1.1)	0.9 ( ± 1.01)	0.04 (± 0.2)	< 0.00001	< 0.00001	< 0.00001
**Severity**	2.1 (± 0.9)	1.1 (± 1.2)	0 (± 0)	< 0.00001	< 0.00001	< 0.00001
**Total score**	8.7 (± 2.8)	4.5 (± 3.7)	0.6 (± 1.1)	< 0.00001	< 0.00001	< 0.00001

ASD, Autism Spectrum Disorder; TS, Tourette Syndrome; TD, typically developing; USP-SPS, University of Sao Paulo’s Sensory Phenomena Scale; p-values refer to to Student’s t tests conducted to compare USP-SPS rating scores between ASD, TS and TD groups (summarized by means and SD).

### Correlations between sensory phenomena and other symptoms

Considering the TS cohort, The USP-SPS total score had significant positive correlations with the CYBOCS total score (r = 0.3015, p = 0.0035) and the MASC total score (r = 0.2365, p = 0.0232). The other relationship between the USP-SPS total scores and the other rating scales did not reach significance (**Table 5**). Conversely, in the ASD group the USP-SPS total score was significantly negative correlated with the total IQ (r = -0.2816, p = 0.0106) and marginally positive correlated with ADOS total score (r = 0.217, p = 0.0502). Instead, the other relationship between the USP-SPS total scores and the other rating scales did not reach statistical significance in the ASD cohort (**Table 5**).

**Table 5:** Pearson’s correlation between sensory phenomena and other symptoms.

TS cohort (n= 92)	USP-SPS total scale		ASD cohort (n= 82)	USP-SPS total scale	
	r	p		r	p
**Total IQ**	-0.0436	0.7050	**Total IQ**	-0.2816	0.0106
**Total YGTSS**	0.0999	0.3478	**Total YGTSS**	0.0343	0.7597
**Total CYBOCS**	0.3015	0.0035	**Total CYBOCS**	0.1736	0.1188
**Total CPRS**	0.1001	0.3424	**Total CPRS**	0.1483	0.1836
**Total CBCL**	0.0294	0.7809	**Total CBCL**	0.127	0.2555
**Total MASC**	0.2365	0.0232	**Total MASC**	0.0493	0.6605
**Total CDI**	0.0294	0.7809	**Total CDI**	-0.1032	0.3571
**Total ADOS**	0.1857	0.07635	**Total ADOS**	0.217	0.0502

ASD, Autism Spectrum Disorder; TS, Tourette Syndrome; USP-SPS, University of Sao Paulo’s Sensory Phenomena Scale.

## Discussion

This study investigates differences between sensory behaviours in TS patients versus ASD patients, compared with a paediatric control sample, through the administration of USP-SPS. So far, a few studies have evaluated sensory phenomena using USP-SPS. Most literature studies were conducted on OCD and/or TS subjects (26–37). Preliminary results of a study conducted on an adult cohort of OCD patients suggested the reliability of USP-SPS for the assessment of sensory behaviours (26). SP were present in 51 OCD patients (67.1%), with a mean USP-SPS total score of 5.5 (SD ± 4.6); among the entire cohort, 16 subjects also presented tics (21.1%), and 13/16 (81.3%) of them presented sensory behaviours (26). Furthermore, tics were twice as common in the patients with SP, but this difference did not reach statistical significance (26). Lee et al. (27) explored the interaction between SP and OCD and showed that all subtypes of SP were significantly more common and severe in OCD than in controls subjects (27). Another study conducted by Sutherland Owens et al. (37) in 18 TS subjects and 22 healthy controls showed a statistically significant positive correlation between USP-SPS and Premonitory Urge for Tics Scale (PUTS) scores in TS subjects (37). In this cohort, USP-SPS total scores tended to grow with age, in line with the clinical experience regarding the age-dependent reporting of premonitory events (38). In a big cross-sectional study that reported data on a large OCD sample, SP were reported in the 72% of the total sample (29). Moreover, compared to OCD patients without comorbid tics, OCD patients affected also by tic disorders showed a higher rate of SP (80.1% vs. 68.6%), but the difference in USP-SPS score was not significant (29). In another study, 1001 OCD patients were assessed to evaluate OCD, tics, comorbidities, level of insight and SP (28). In this sample, 651 (65.0%) presented at least one subtype of SP associated to repetitive behaviours (28). The comparison of OCD patients with and without SP showed also a significantly more common comorbid diagnosis of TS and Chronic tic disorders (CTD), and a positive family history of tic disorders in the OCD group with SP, that in the other group (28). In addition, the same author’s group compared the subtypes of SP in OCD participants with and without tics and showed that patients affected by OCD associated with tic disorders endorsed SP significantly more frequently than OCD patients without tics (31). In 2014, Sampaio et al. conducted a study to validate and investigate the psychometric properties of the English version of the USP-SPS (30). In this study, SP were detected in 89.1% of OCD sample, and 100% of patients with tic disorders, supporting the high presence of SP in OCD and TS sample (30). In another study, a structural correlate of SP involving grey matter volume increases within the sensorimotor cortex was identified in patients with OCD (33), in line with the results reported in another study conducted in patients with tic disorders, showing abnormal activity and volume increases within this region are associated with the urges preceding tic onset (39). Furthermore, another study investigated such phenomena associated to tics, obsessive-compulsive symptoms (OCS), and global functioning in a small sample of TS patients (32). The authors reported a significant correlation between the PUTS and the USP-SPS total score; in addition, USP-SPS and PUTS total scores were significantly correlated with YGTSS total scores and Dimensional Yale-Brown Obsessive-Compulsive Scale (DY-BOCS) total scores (32). Moreover, de Avila et al. (34) investigated factors associated with poor insight in subjects with OCD and demonstrated that patients with poor insight differed from those with good insight regarding more prevalent SP (34). In addition, another recent report on a small TS sample by the same author’s group described changes in SP, tics, OCD after 4 years (35). A significantly correlation between previous USP-SPS and PUTS total scores and previous YGTSS and Y-BOCS total scores was revealed, while current USP-SPS total scores were significantly correlated with current YGTSS global severity scores (35). Additionally, current USP-SPS and PUTS total scores were significantly correlated with current YBOCS total scores, while previous USP-SPS total scores were significantly correlated with current Y-BOCS total scores and marginally correlated with current YGTSS global severity scores (35). Recently, Vellozo et al. (36) compared OCD patients with and without symptoms of the symmetry dimension to evaluate their clinical profiles and reported that the OCD group with symmetry symptoms presented higher frequency and severity of SP (36) Previous studies regarding the assessment of SP using USP-SPS in TS and/or OCD patients are summarized in **Table 6**.

**Table 6:** Summary of studies regarding SP using USP-SPS in TS and/or OCD patients.

Reference	Patients (n)	Mean age	Comorbidities	USP-SPS Total Score	Results
Rosario et al. (26)	76 OCD	35.4	-Tics (21.1%)	5.5 (SD = 4.6)	SP were present in 67.1% of patients. There were no significant differences in the presence of SP according to comorbidity with tics.
Lee et al. (27)	37 OCD	37	-Tic disorders (20%) -OCPD (35%)	n.a.	The frequency of any kind of SP was significantly higher in OCD patients (67.6%) when compared to controls (35.1%).
Sutherland Owens et al. (37)	18 TS	9 adults (25.5) 9 children (13.2)	-OCD (50%) -ADHD (11.1%)	8.5 (SD = 3.7)	Statistically significant positive correlation between USP-SPS and PUTS total scores.
Gomes de Alvarenga et al. (29)	813 OCD	34.9	-Tic disorders (29.0%) -Mood disorders (70.7%) -Anxiety disorders (33.8%) -ADHD (16.1%) -Impulsive control disorders (37.3%) -Body dysmorphic disorders (11.9%) -Others	37.08	SP were reported by 72% of the entire sample. Compared to OCD patients without comorbid tics, OCD patients with comorbid tic disorders were more likely to present SP.
Ferrão et al. (28)	1001 OCD	34.85	-TS (8.8%) -CTD (13.7%) -Trichotillomania (4.5%) -Skin picking (15.4%)	7.7 (SD = 3.49)	651 (65.0%) subjects reported at least one type of SP preceding the repetitive behaviours. The presence of SP was associated with comorbid TS, and a family history of tic disorders.
Shavitt et al. (31)	1001 OCD	34.85	-TS (9%) -CTD (15.4%) -Mood disorders (42.6%) -Anxiety disorders (65%) -Impulse control disorders (30.8%) -ADHD (12.7%) -Others	4.88 (SD = 4.63)	Most OCD patients endorsed SP (60.4%). OCD + TS and OCD + CTD endorsed SP significantly more frequently than OCD patients without tics.
Sampaio et al. (30)	60 OCD and/or TS	18.98	-OCD (91.7%) -TS (26.7%) -CTD (5%)	n.a.	The prevalence of SP in total sample was 88.5%. SP were presented in 89.1% of OCD sample, and 100% of TS and CTD sample.
Subirà et al. (33)	106 OCD	33.11	n.a.	8.4 (SD = 3.5)	Patients with SP (67%) showed grey matter volume increases in the left sensorimotor cortex in comparison to Patients without SP and bilateral sensorimotor cortex grey matter volume increases in comparison to controls.
Kano et al. (32)	41 TS	23.1	-OCD (20%)	6.4 (SD = 3.1)	The PUTS total score had significant correlations with the USP-SPS total score. USP-SPS and PUTS total scores were significantly correlated with YGTSS total scores and DY-BOCS total scores.
de Avila et al. (34)	272 OCD	Poor insight (n= 124, median 35.5), Good insight (n=148, median 32)	-Tics (median 34 in poor insight, 41 in good insight); -TS (median 10 in poor insight, 9 in good insight); -ADHD (median 22 in poor insight, 14 in good insight); -Others	9 (median)	Individuals affected by OCD in the poor insight group presented more prevalent SP compared to those with good insight.
Kano et al. (35)	20 TS	30.2	-OCD (30%) -ADHD (20%)	5.0 (SD = 3.2)	Current USP-SPS total scores were significantly correlated with current YGTSS global severity scores. Both current USP-SPS total scores and PUTS total scores were significantly correlated with current CY-BOCS total scores.
Vellozo et al. (36)	1001 OCD	34.8	-TS (8.8%) -Tic disorders (28.4%) -ADHD (13.7%) -Mood disorders (60.8%) -Anxiety disorders (69.8%) -Others	4.9 (SD = 4.6)	The OCD group with symmetry symptoms presented higher frequency and severity of SP.

SP, sensory phenomena; USP-SPS, University of Sao Paulo’s Sensory Phenomena Scale; TS, Tourette Syndrome; OCD, obsessive-compulsive disorder; SD, standard deviation; OCPD, Obsessive compulsive personality disorder; ADHD, Attention-deficit hyperactivity disorder; PUTS, Premonitory Urge for Tics Scale; CTD, Chronic tic disorders; n.a., not available; DY-BOCS, Dimensional Yale-Brown Obsessive-Compulsive Scale; CY-BOCS, Children’s Yale-Brown Obsessive-Compulsive Scale for Children.

The results of our study show that SP were present in 76 TS patients (82.6%), 82 ASD patients (100%) and 31 TD subjects (31%). In the TS cohort, the mean USP-SPS total score was slightly lower (mean 4.5, SD ± 3.7) respect to other reported samples (32, 35, 37). Furthermore, the most frequently reported types of SP in the TS cohort are tactile physical sensations (58.7%) and look “just-right” perceptions (51.1%). Other studies conducted on TS samples by the same author’s group (32, 35) reported a higher frequency of muscle-joint physical sensations, tactile “just-right” perception, and urge only. The differences detected between our results and other literature studies are probably due to the different range of age of other reported cohorts, that included more adult TS patients, respect to our paediatric sample. In addition, a broader spectrum of comorbidities was described in our sample, compared to other literature studies, that reported cohorts of TS patients with a concomitant diagnosis of ADHD and/or OCD (32, 35, 37).

In this study, we detected a significant positive correlation between the USP-SPS total score and the CYBOCS total score (r = 0.0909, p = 0.0035), in line with previous results (32, 35). There results suggested that both tics and OCD symptoms have strong relationships with SP, in line with other reports. Instead, there are few data available regarding the assessment of SP in TS cohort through the USP-SPS. Further studies are needed to better characterize these kinds of phenomena in patients with tic disorders. To the best of our knowledge, this is the first study in which SP were assessed administering the USP-SPS scale in ASD cohort. Considering the psychometric properties of USP-SPS for the assessment of presence and severity of SP, further research is required to understand the complexity of these kind of phenomena in larger ASD cohorts. Conversely, several studies have focused on the characterization of SP in children with ASD, using other instruments (40, 41). In our ASD cohort, hypersensitivity was the type of SP most represented (98.8%), in line with literature studies that reported a higher prevalence of sensory over-responsivity (SOR) involving different sensory modalities (42). Furthermore, tactile physical sensations (82.9%) and look “just-right” perceptions (75.6%) are more frequent in our ASD-group. Of note, atypicality in visual and tactile processing were frequently reported as a typical sensory difficulty in children with ASD (43). Certainly, it would be desirable to make a thorough assessment of SP in ASD, comparing USP-SPS with other tools evaluating abnormalities in sensory processing. Our results show that SP are more frequently reported in ASD cohort than TS population. Furthermore, unusual sensory behaviours have been described for other neurodevelopmental disorders, but they are particularly frequent in individuals with ASD, with about 90% of autistic individuals presenting an atypical sensory profile and with an elevated variability among individual sensory modalities (44). Given the higher rate of sensory processing abnormalities in ASD, sensory abnormalities were added as core diagnostic features of ASD in DSM-5 (1).

Several limitations in our study must be discussed. First, larger cohorts would be needed to improve our knowledge on the differences in sensory behaviours between ASD and TS. Second, complementing the assessment of SP with other questionnaires could be useful to more characterize the phenomenology of SP. Third, considering that the recruitment was done in a tertiary centre, it may be argued that only moderate to severe patients were included in the study. In addition, it is important to underline that most patients recruited were not affected only by TS or ASD, but presented associated comorbid psychopathologies. Furthermore, it would be helpful to explore the possible influence of associated comorbidities on the prevalence of SP in children with TS and/or ASD, with particular reference to OCD, taking into account the results reported in the literature studies conducted on OCD samples. Due to these limitations, further investigations that evaluating SP using USP-SPS in TS and ASD groups would be meaningful, considering the paucity of literature reports on paediatric cohorts.

## Conclusions

This study highlights that SP are a common characteristic both of ASD and TS. Considering the heterogeneity of these conditions, a more detailed exploration of the SP and their subtypes could help to better understanding the differences on their phenomenology in patients with TS and ASD. Future studies should include the application of tools such as USP-SPS that evaluate these phenomena in larger paediatric cohorts of patients with ASD and TS, also exploring the possible impact of comorbid conditions.

## Data availability statement

The raw data supporting the conclusions of this article will be made available by the authors, without undue reservation.

## Ethics statement

The studies involving humans were approved by Local Ethics Committee (Catania 1) of Catania University Hospital. The studies were conducted in accordance with the local legislation and institutional requirements. Written informed consent for participation in this study was provided by the participants’ legal guardians/next of kin.

## Author contributions

AP: Data curation, Formal analysis, Writing – original draft. FS: Data curation, Formal analysis, Writing – original draft. MF: Data curation, Writing – original draft. VF: Data curation, Writing – original draft. RB: Methodology, Writing – review & editing. RR: Conceptualization, Methodology, Supervision, Writing – review & editing.

## References

[1] American-Psychiatric-Association. Diagnostic and statistical manual of mental disorders. Washington, DC, USA: American Psychiatric Publishing (2013).

[2] Robertson MM Eapen V Singer HS Martino D Scharf JM Paschou P. Gilles de la Tourette syndrome. Nat Rev Dis Primers. (2017) 3:16097. doi: 10.1038/nrdp.2016.97 28150698

[3] Hirschtritt ME Lee PC Pauls DL Dion Y Grados MA Illmann C. Lifetime prevalence, age of risk, and genetic relationships of comorbid psychiatric disorders in Tourette syndrome. JAMA Psychiatry. (2015) 72:325–33. doi: 10.1001/jamapsychiatry.2014.2650 PMC4446055 25671412

[4] Cravedi E Deniau E Giannitelli M Xavier J Hartmann A Cohen D. Tourette syndrome and other neurodevelopmental disorders: a comprehensive review. Child Adolesc Psychiatry Ment Health. (2017) 11:59. doi: 10.1186/s13034-017-0196-x 29225671 PMC5715991

[5] Prado H do Rosário MC Shavitt RG Miguel EC. Sensory phenomena, "just-right" and "not just-right" experiences in OCD patients: looking for a consensus. CNS spectrums. (2007) 12:95–6. doi: 10.1017/S1092852900020587 17375447

[6] Lord C Brugha TS Charman T Cusack J Dumas G Frazier T. Autism spectrum disorder. Nature reviews. Dis Primers. (2020) 6:5. doi: 10.1038/s41572-019-0138-4 PMC8900942 31949163

[7] Maenner MJ Shaw KA Baio J Washington A Patrick M DiRienzo M. Prevalence of autism spectrum disorder among children aged 8 years - autism and developmental disabilities monitoring network, 11 sites, United States 2016. Morbidity Mortality Weekly Rep Surveillance Summaries (Washington D.C.: 2002). (2020) 69:1–12. doi: 10.15585/mmwr.ss6904a1 PMC7119644 32214087

[8] Schaaf RC Case-Smith J. Sensory interventions for children with autism. J Comp Effectiveness Res. (2014) 3:225–7. doi: 10.2217/cer.14.18 24969147

[9] Tavassoli T Bellesheim K Tommerdahl M Holden JM Kolevzon A Buxbaum JD. Altered tactile processing in children with autism spectrum disorder. Autism Research: Off J Int Soc Autism Res. (2016) 9:616–20. doi: 10.1002/aur.1563 26568449

[10] Ben-Sasson A Hen L Fluss R Cermak SA Engel-Yeger B Gal E. A meta-analysis of sensory modulation symptoms in individuals with autism spectrum disorders. J Autism Dev Disord. (2009) 39:1–11. doi: 10.1007/s10803-008-0593-3 18512135

[11] Lane AE Young RL Baker AE Angley MT. Sensory processing subtypes in autism: association with adaptive behaviour. J Autism Dev Disord. (2010) 40:112–22. doi: 10.1007/s10803-009-0840-2 19644746

[12] Robertson MM. A personal 35-year perspective on Gilles de la Tourette syndrome: prevalence, phenomenology, comorbidities, and coexistent psychopathologies. Lancet Psychiatry. (2015) 2:68–87. doi: 10.1016/S2215-0366(14)00132-1 26359614

[13] Burd L Li Q Kerbeshian J Klug MG Freeman RD. Tourette syndrome and comorbid pervasive developmental disorders. J Child Neurol. (2009) 24:170–5. doi: 10.1177/0883073808322666 19182154

[14] Darrow SM Grados M Sandor P Hirschtritt ME Illmann C Osiecki L. Autism spectrum symptoms in a tourette's disorder sample. J Am Acad Child Adolesc Psychiatry. (2017) 56:610–617.e1. doi: 10.1016/j.jaac.2017.05.002 28647013 PMC5648014

[15] Gulisano M Barone R Mosa MR Milana MC Saia F Scerbo M. Incidence of Autism Spectrum Disorder in Youths Affected by Gilles de la Tourette syndrome based on Data from a Large Single Italian Clinical Cohort. Brain Sci. (2020) 10:812. doi: 10.3390/brainsci10110812 33147879 PMC7692268

[16] Rapanelli M Frick LR Pittenger C. The role of interneurons in autism and tourette syndrome. Trends Neurosci. (2017) 40:397–407. doi: 10.1016/j.tins.2017.05.004 28578790 PMC5528854

[17] Rutter M Bailey A Lord C. Social communication questionnaire. Los Angeles, CA: Western Psychological Services (2003).

[18] Wechsler D. Wechsler intelligence scale for children. New York, NY: The Psychological Corporation (1949).

[19] Leckman JF Riddle MA Hardin MT Ort SI Swartz KL Stevenson J. The Yale Global Tic Severity Scale: initial testing of a clinician-rated scale of tic severity. J Am Acad Child Adolesc Psychiatry. (1989) 28:566–73. doi: 10.1097/00004583-198907000-00015 2768151

[20] Scahill L Riddle MA McSwiggin-Hardin M Ort SI King RA Goodman WK. Children's Yale-Brown Obsessive Compulsive Scale: reliability and validity. J Am Acad Child Adolesc Psychiatry. (1997) 36:844–52. doi: 10.1097/00004583-199706000-00023 9183141

[21] Lord C Risi S Lambrecht L Cook EH Leventhal BL DiLavore PC. The autism diagnostic observation schedule-generic: a standard measure of social and communication deficits associated with the spectrum of autism. J Autism Dev Disord. (2000) 30:205–23. 11055457

[22] Conners C. Conners’ Rating Scales–Revised technical manual. North Tonawanda, NY: Multi-Health Systems (1997). Available at: http://www.mhs.com.

[23] Achenbach T Edelbrock C. The child behaviour checklist manual. Burlington, VT: The University of Vermont (1991).

[24] Kovacs M. The children’s depression inventory: a self-rated depression scale for school aged youngsters (Italian version). Firenze: OrganizzazioniSpeciali. (1988).

[25] March JS Parker JD Sullivan K Stallings P Conners CK. The Multidimensional Anxiety Scale for Children (MASC): factor structure, reliability, and validity. J Am Acad Child Adolesc Psychiatry. (1997) 36:554–65. doi: 10.1097/00004583-199704000-00019 9100431

[26] Rosario MC Prado HS Borcato S Diniz JB Shavitt RG Hounie AG. Validation of the University of São Paulo Sensory Phenomena Scale: initial psychometric properties. CNS spectrums. (2009) 14:315–23. doi: 10.1017/s1092852900020319 19668122

[27] Lee JC Prado HS Diniz JB Borcato S da Silva CB Hounie AG. Perfectionism and sensory phenomena: phenotypic components of obsessive-compulsive disorder. Compr Psychiatry. (2009) 50:431–6. doi: 10.1016/j.comppsych.2008.11.007 19683613

[28] Ferrão YA Shavitt RG Prado H Fontenelle LF Malavazzi DM de Mathis MA. Sensory phenomena associated with repetitive behaviours in obsessive-compulsive disorder: an exploratory study of 1001 patients. Psychiatry Res. (2012) 197:253–8. doi: 10.1016/j.psychres.2011.09.017 22361443

[29] Gomes de Alvarenga P de Mathis MA Dominguez Alves AC do Rosário MC Fossaluza V Hounie AG. Clinical features of tic-related obsessive-compulsive disorder: results from a large multicenter study. CNS Spectrums. (2012) 17:87–93. doi: 10.1017/S1092852912000491 22789066

[30] Sampaio AS McCarthy KD Mancuso E Stewart SE Geller DA. Validation of the University of São Paulo’s sensory phenomena scale – english version. Compr Psychiatry. (2014) 55:1330–6. doi: 10.1016/j.comppsych.2014.02.008 24666717

[31] Shavitt RG de Mathis MA Oki F Ferrao YA Fontenelle LF Torres AR. Phenomenology of OCD: lessons from a large multicenter study and implications for ICD-11. J Psychiatr Res. (2014) 57:141–8. doi: 10.1016/j.jpsychires.2014.06.010 PMC7326117 25012187

[32] Kano Y Matsuda N Nonaka M Fujio M Kuwabara H Kono T. Sensory phenomena related to tics, obsessive-compulsive symptoms, and global functioning in Tourette syndrome. Compr Psychiatry. (2015) 62:141–6. doi: 10.1016/j.comppsych.2015.07.006 26343478

[33] Subirà M Sato JR Alonso P do Rosário MC Segalàs C Batistuzzo MC. Brain structural correlates of sensory phenomena in patients with obsessive-compulsive disorder. J Psychiatry Neurosci JPN. (2015) 40:232–40. doi: 10.1503/jpn.140118 PMC4478056 25652753

[34] de Avila RCS do Nascimento LG Porto RLM Fontenelle L Filho ECM Brakoulias V. Level of insight in patients with obsessive-compulsive disorder: an exploratory comparative study between patients with "Good insight" and "Poor insight". Front Psychiatry. (2019) 10:413. doi: 10.3389/fpsyt.2019.00413 31333508 PMC6619338

[35] Kano Y Fujio M Kaji N Matsuda N Nonaka M Kono T. Changes in sensory phenomena, tics, obsessive-compulsive symptoms, and global functioning of tourette syndrome: A follow-up after four years. Front Psychiatry. (2020) 11:619. doi: 10.3389/fpsyt.2020.00619 32695033 PMC7338586

[36] Vellozo AP Fontenelle LF Torresan RC Shavitt RG Ferrão YA Rosário MC. Symmetry dimension in obsessive-compulsive disorder: prevalence, severity and clinical correlates. J Clin Med. (2021) 10:274. doi: 10.3390/jcm10020274 33451078 PMC7828517

[37] Sutherland Owens AN Miguel EC Swerdlow NR. Sensory gating scales and premonitory urges in Tourette syndrome. Sci World J. (2011) 11:736–41. doi: 10.1100/tsw.2011.57 PMC5548288 21442151

[38] Woods DW Piacentini J Himle MB Chang S. Premonitory Urge for Tics Scale (PUTS): initial psychometric results and examination of the premonitory urge phenomenon in youths with Tic disorders. J Dev Behav Pediatrics: JDBP. (2005) 26:397–403. doi: 10.1097/00004703-200512000-00001 16344654

[39] Draganski B Martino D Cavanna AE Hutton C Orth M Robertson MM. Multispectral brain morphometry in Tourette syndrome persisting into adulthood. Brain: J Neurol. (2010) 133:3661–75. doi: 10.1093/brain/awq300 PMC2995885 21071387

[40] Simpson K Adams D Alston-Knox C Heussler HS Keen D. Exploring the sensory profiles of children on the autism spectrum using the short sensory profile-2 (SSP-2). J Autism Dev Disord. (2019) 49:2069–79. doi: 10.1007/s10803-019-03889-2 30673910

[41] Barrios-Fernández S Gozalo M Díaz-González B García-Gómez A. A complementary sensory tool for children with autism spectrum disorders. Children (Basel Switzerland). (2020) 7:244. doi: 10.3390/children7110244 33233607 PMC7699787

[42] Baranek GT Boyd BA Poe MD David FJ Watson LR. Hyperresponsive sensory patterns in young children with autism, developmental delay, and typical development. Am J Ment Retardation: AJMR. (2007) 112:233–45. doi: 10.1352/0895-8017(2007)112[233:HSPIYC]2.0.CO;2 17559291

[43] Narzisi A Fabbri-Destro M Crifaci G Scatigna S Maugeri F Berloffa S. Sensory profiles in school-aged children with autism spectrum disorder: A descriptive study using the sensory processing measure-2 (SPM-2). J Clin Med. (2022) 11:1668. doi: 10.3390/jcm11061668 35329994 PMC8955781

[44] Scheerer NE Curcin K Stojanoski B Anagnostou E Nicolson R Kelley E. Exploring sensory phenotypes in autism spectrum disorder. Mol Autism. (2021) 12(1):67. doi: 10.1186/s13229-021-00471-5 34641960 PMC8507349

